# Walking speed-related changes in stride time variability: effects of decreased speed

**DOI:** 10.1186/1743-0003-6-32

**Published:** 2009-08-05

**Authors:** Olivier Beauchet, Cedric Annweiler, Yhann Lecordroch, Gilles Allali, Veronique Dubost, François R Herrmann, Reto W Kressig

**Affiliations:** 1 Department of Internal Medicine and Geriatrics, Angers University Hospital, UPRES UNAM EA 2646, University of Angers, UNAM, France; 2 Department of Rehabilitation & Geriatrics, Geneva University Hospitals, Switzerland; 3 Department of Neurology, Geneva University Hospitals, Switzerland; 4 FORMADEP, Korian, France; 5 Department of Geriatrics, Basel University Hospital, Switzerland

## Abstract

**Background:**

Conflicting results have been reported regarding the relationship between stride time variability (STV) and walking speed. While some studies failed to establish any relationship, others reported either a linear or a non-linear relationship. We therefore sought to determine the extent to which decrease in self-selected walking speed influenced STV among healthy young adults.

**Methods:**

The mean value, the standard deviation and the coefficient of variation of stride time, as well as the mean value of stride velocity were recorded while steady-state walking using the GAITRite^® ^system in 29 healthy young adults who walked consecutively at 88%, 79%, 71%, 64%, 58%, 53%, 46% and 39% of their preferred walking speed.

**Results:**

The decrease in stride velocity increased significantly mean values, SD and CoV of stride time (p < 0.001), whereas the repetition of trials (p = 0.534, p = 0.177 and p = 0.691 respectively for mean, SD, CoV); and step asymmetry (p = 0.971, p = 0.150 and p = 0.288 for mean, SD and CoV) had no significant effect. Additionally, the subject's effect was significant for all stride parameters (p < 0.001). The relationship between a decrease in walking speed and all stride parameters (i.e., mean values, SD and CoV of stride time) was significantly quadratic and showed higher STV at a slow speed (p < 0.001).

**Conclusion:**

The results support the assumption that gait variability increases while walking speed decreases and, thus, gait might be more unstable when healthy subjects walk slower compared with their preferred walking speed. Furthermore, these results highlight that a decrease in walking speed can be a potential confounder while evaluating STV.

## Background

Human walking is an automated rhythmic motor behavior [1-3]. Automaticity and rhythmicity imply that a healthy subject is able to reproduce comparable limb-coordinated movements from stride-to-stride while steady state walking [2,3]. Stride-to-stride variability is a measure of the consistency of limb movements [2]. In particular, stride time variability [STV], as calculated out off the mean and standard deviation [SD] of stride time and expressed as the coefficient of variation [CoV], is a measure of temporal stride kinematic variability related to the control of the rhythmic stepping mechanism. Low variability values of stride time reflect the automated regular rhythmic feature of gait and are associated with safe gait and are used as a clinical index of gait stability [4-9]. Because walking is one of the most repetitive and "hard wired" human movements, STV is low and usually below 3% among young healthy adults [9-12].

Changes in gait variability outside of the normal range of gait variability must be studied cautiously as several factors may influence STV. Although the relation between an increased STV and certain neuro-degenerative diseases is well-established [i.e., Parkinson's and Alzheimer's disease], the possible effect of a decrease in walking speed on STV has been rarely examined and is still controversially discussed [13-17]. Previous studies obtained conflicting results, as some failed to find any relationship [12,13] while others reported either a linear or a non-linear relationship [14-17]. Additionally, most of these studies did not take into account the effects of potential confounders that may modify the STV, such as between-subjects variability, the repetition of trials, left-right step asymmetry or the use of motorized treadmills [6,7,12-17]. It is therefore unclear whether an increase in STV is provoked either by the decrease in walking speed, by confounders, or both.

The aim of this study was to determine the extent to which a decrease in self-selected walking speed influenced STV among healthy young adults.

## Methods

### Subjects

Twenty-nine young adults (15 men and 14 women; mean age 28.3 ± 6.2 years; range: 18–39 years) were recruited after having given their informed consent. The participants reported no physical or mental disorders. They were not taking any medication. The study was approved by the local Ethics Committee and conducted in accordance with the ethical standards set forth in the Declaration of Helsinki (1983).

### Tasks and procedure

Participants were asked to straight walk in non-randomized order and consecutively at 80%, 70%, 60%, 50%, 40%, 30%, 20% and 10% of their preferred walking speed. Self-selected speed was freely chosen following the instructions of evaluator to reduce the preferred walking speed by 10% percentage. The subjects were instructed that they should reduce their walking speed from their preferred walking speed to 10% of it. The verbal instructions were standardized: "You will straight walking at your self selected walking speed. You will start at your preferred walking speed and you will decrease your walking speed stage by stage of 10% until walking to 10% of your preferred walking speed. You will perform 3 trials for each walking speed condition. Have you understood the instructions? Do you have any question about this test?" All subjects started gait recording with their preferred walking speed. Because the design was based on freely chosen walking speed, the real speed decreasing rate differed from the theoretical rate, and was therefore calculated using the following formula: [(measured self-selected stride velocity/preferred stride velocity) × 100]. The results showed that participants actually walked at 89%, 80%, 72%, 65%, 58%, 53%, 46% and 39% of their preferred walking speed. Participants completed 3 trials for each level of decrease in walking speed. Before the test was carried out, a trained evaluator gave standardized verbal instructions regarding the test procedure with a visual demonstration of the walking test. The walking trials were carried out in a well-lit environment, with subjects wearing their own footwear. According to the guidelines for spatio-temporal gait analysis, and in order to ensure that gait parameters were collected while steady state walking, participants started walking at least 2 meters before reaching the electronic walkway and completed their walk at least two meters beyond it [18].

### Apparatus

The GAITRite^®^-System (GAITRite Gold, CIR Systems, PA, USA) is an electronic walkway-integrated, pressure-sensitive electronic surface of 7.32 × 0.61 m, connected to a portable computer via an interface cable [7]. The carpet includes a series of sensors (a total of 13824 sensors) placed every 1.27 cm with their centers placed in a 48 × 288 grid and activated by mechanical pressure. The data from the activated sensors is collected by a series of on-board processors and transferred to the computer through a serial port. The data is sampled from the carpet at a frequency of 80 Hz, allowing a temporal resolution of 12.5 ms. Stride time (i.e. gait cycle duration) is defined as the time elapsed between the first contact of two consecutive footsteps of the same foot and is expressed in milliseconds.

### Outcomes

The following outcome variables were used: Mean value and SD expressed in second and CoV of stride time (CoV = [(SD/mean) × 100] expressed in percentage; and Mean value of stride velocity for each walking condition expressed in cm.s^-1^. The primary outcome measure was CoV of stride time. Mean value and SD of stride time, and Mean value of stride velocity were the secondary outcomes measures. Repetitions of trial, subjects' effect and step asymmetry were used as covariates in data analysis.

### Data analysis

Stride time and stride velocity values were summarized using means and standard deviations. The normality of the parameters' distribution was verified with skewness and kurtosis tests before and after applying usual transformations to try to normalize non-Gaussian variables. As these transformations were unable to achieve normalization of the distribution, raw values had to be used. Firstly, a comparison of the outcomes, based on the Kruskal-Wallis test, was carried out for the 3 trials of each walking condition performed. The aim was to convert the three trials into one single trial if no significant difference between trials was observed. The Spearman Brown prophecy coefficient was used to estimates test-retest reliability of the 3 trials. Secondly, a comparison of stride velocity for each level of decrease in walking speed was performed using the Cuzick test. Thirdly, a balanced repeated measures analysis of variance (ANOVA) was performed in order to estimate the effects of a decrease in walking speed on the mean value, SD and CV of stride time while adjusting for the 3 trial repetitions, for each walking condition and for subjects' effect, taking account of the variability between subjects and step asymmetry without interactions terms. Fourthly, a univariate quadratic regression was performed to separately explore the association between decreased walking speed and mean value, SD, and CoV of stride time, respectively; the preferred walking speed served as reference level. *P *< 0.05 was considered statistically significant. Our statistics were calculated using the Stata Statistical Software, release 9.2 [19].

## Results

**Figure 1:**
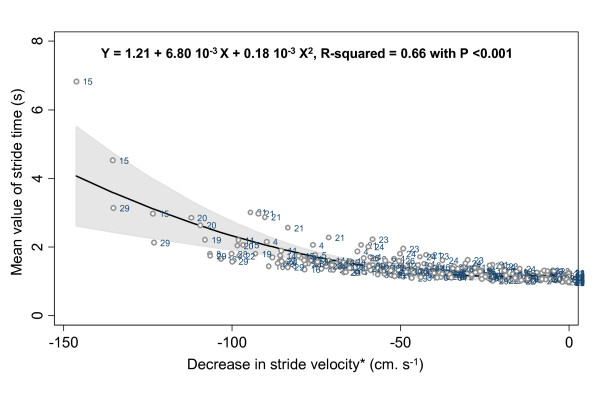
**Quadratic regression inquiring into a possible association between mean value of stride time and decrease in self-selected walking speed, with the reference value set as the normal self-selected walking speed among young healthy adults (n = 29)**. *: Normal self-selected walking speed used as the reference level and coded as 0 cm.s^-1^.

**Figure 2:**
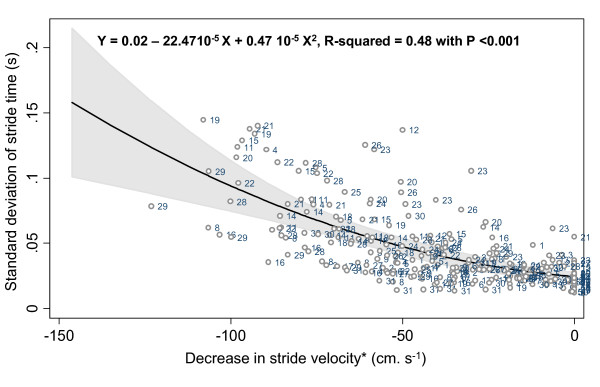
**Quadratic regression inquiring into a possible association between standard deviation of stride time and decrease in self-selected walking speed, with the reference value set as the normal self-selected walking speed among young healthy adults (n = 29)**. *: Normal self-selected walking speed used as the reference level and coded as 0 cm.s^-1^.

**Figure 3:**
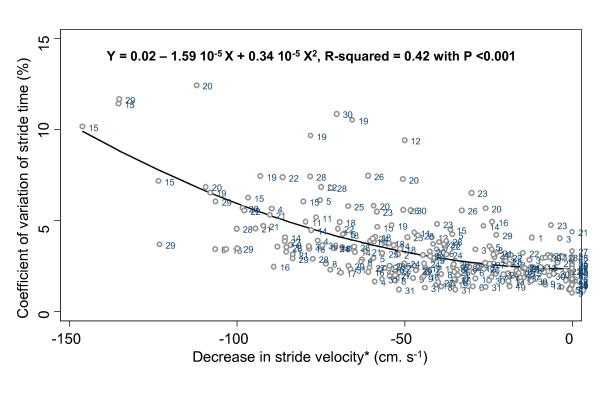
**Quadratic regression inquiring into a possible association between coefficient of variation of stride time and decrease in self-selected walking speed, with the reference value set as the normal self-selected walking speed among young healthy adults (n = 29)**. *: Normal self-selected walking speed used as the reference level and coded as 0 cm.s^-1^.

The mean values and SD of stride time and stride velocity parameters are summarized in a Table [See additional file 1]. There was no significant difference for all stride parameters between the 3 trials for each level of decrease in walking speed. The stride velocity decreased significantly from 88 to 39% of the preferred waking speed (p-trend < 0.001). The ANOVA model (Table 1). showed that the decrease in stride velocity explained the increase in mean values, SD and CoV of stride time (p < 0.001), whereas the repetition of trials (p = 0.534, p = 0.177 and p = 0.691 respectively for mean, SD, CoV); and step asymmetry (p = 0.971, p = 0.150 and p = 0.288 for mean, SD and CoV) had no significant effect. The estimated trial reliability amounted to 96.3% for the mean, 93.1% for the SD and 89.9% for the CV of stride time. Additionally, the subject's effect was significant for all stride parameters (p < 0.001). As shown in figures 1, 2 and 3, the relationship between a decrease in walking speed and stride parameters was quadratic and showed higher STV at a slow speed (p < 0.001).

**Table 1:** *P*-value of repeated measures analysis of variance (ANOVA) (n = 1280 steps) estimating the effects of a decrease in self-selected walking speed on mean value, standard deviation and coefficient of variation of stride time, adjusted for subject's effect (n = 29), number of trials per walking condition and left-right step asymmetry

	Effect				
		
Stride time	Decrease in preferred walking speed	Subject	Trials*	Left-right step asymmetry†	R-squared
Mean value	<0.001	<0.001	0.534	0.971	0.612
SD	<0.001	<0.001	0.177	0.150	0.481
CoV	<0.001	<0.001	0.691	0.288	0.293

SD: standard deviation,

CoV: Coefficient of variation expressed in percentage and calculated from the formula: [(Standard deviation/Mean value) × 100],

*: Number of trials per walking condition coded in three level (0 = first trial, 1 = second trial, 2 = third trial)

†: Right and left step coded as a binary variable (0 = Right, 1 = Left)

## Discussion

Our results show that STV increased while walking speed decreased, even when taking into account an adjustment for the subjects' effect, the repetition of trials and the left-right step asymmetry. This finding has two main implications. Firstly, gait may become more unstable as walking speed decreases. Secondly, the decrease in walking speed should be considered as a potential confounder while evaluating STV in gait disorders associated with a decrease in walking speed.

The relationship between STV and walking speed is complex and not fully established, as some studies failed to find any relationship [12,13], while others reported either a linear or a non-linear relationship [14-17]. Our study solely focused on the effects of decreased walking speed, whereas previous studies analyzed both increased and decreased walking speeds, and corroborated earlier data establishing a significant negative correlation between STV and walking speed [14-17]. Only two studies did not find any relationship between gait speed and stride time variability [12,13]. This apparent controversy may result from the fact that only the effect of a low walking speed decrease was examined in these two studies. It has been shown that a higher STV was reported at very slow walking speeds (e.g. 0.2 to 0.6 m.s^-1^), compared to moderated speeds (e.g. 0.8 to 1.4 m.s^-1^)[16]. Subjects walked at a walking speed 10% slower than their preferred speed, corresponding to walking at 0.9 m.s^-1 ^in Owing's study [13] and 1.0 m.s^-1 ^in Frenkel-Toledo's study [12]. Therefore, the decrease in walking speed was probably too small to show a significant increase in STV. Recently, Jordan *et al. *[15] also showed a similar negative association between walking speed and STV. STV decreased while walking speed increased. However, unlike in our study design, both decreased and increased walking speeds were used as a basis to report this significant relationship. Lower STV was only observed with fast walking speed and not with preferred speed.

Our results provide the evidence that the relationship between decreased walking speed and increased STV is not linear but quadratic. Heiderscheit [16] was the first to suggest a U-shaped curved non-linear relationship between STV and walking speed, whereby higher STV was observed at slow and fast speed, whereas lower STV was recorded at preferred speed. However, no statistical analysis was performed to confirm these descriptive results. There are several others arguments in favor of a non-linear relationship, provided by the analysis of walk-run and run-walk transition ranges of walking speed. Brisswalter & Mottet [20] observed a consistent level of variability before and after these transitions. Furthermore, Belli *et al. *[14] also reported that STV significantly increased while walking speed changed from preferred speed to 140% at maximal speed. In addition, these results support the assumption that cyclic movements, like limb-movements while walking, have maximal cycle variability at specific cycle frequencies [3].

In contrast to previous studies [12-17], we took into account the role of potential confounders that may modify the relationship between STV and walking speed. Firstly, the subjects' effect was considered as a variable that may in part explain the increased STV. In order to compute the error term in the ANOVA model, the subjects were nested within walking conditions. The subjects' effect is a specific case of group effect, with each group having only one member; when one deals with repeated measures, meaning that many observations are recorded for the same subjects. The subjects' effect allows adjusting a model for some unknown subject's characteristics and provides information on the heterogeneity among subjects that may artificially increase the STV of a group of subjects. Secondly, the development of a motor skill is possible while walking trials are repeated. Many studies used a motorized treadmill that required participants' training before gait parameters could be measured [12-15]. It has been shown that limb movement variability decreases as a function of practice and increment of skills [1-3]. The training phase or the repetition of trials may therefore reduce STV. In our study, although no treadmill was used, this confounder was examined and had no impact on STV. Thirdly, an increase in STV may be explained by left-right step asymmetry in healthy subjects [13,14]. This confounder was solely controlled for in the study by Owings & Grabiners [13], in which an absence of left-right stride asymmetry in healthy adults was reported, similarly to the results we have obtained. Fourthly, our study used a specific procedure in which the decreased walking speed was freely chosen following the instruction to reduce the normal walking speed by a certain percentage. Because STV could be influenced by the imposed walking speed of a motorized treadmill [1-3], we used a GAITRite^® ^system [7]. This device is an electronic carpet which specifically respects the ecological walking condition by recording the freely chosen walking speed. Freely chosen walking speed is primordial when examining STV because it is the only walking speed that takes into account the strategy aiming at maintaining an optimal index of movement consistency in terms of energy costs, attentional demand and efficiency of gait control, leading to a low STV [5,6,9,20].

Both increased STV and slow walking speed have been independently related to gait instability [4,6,9,11,21]. Therefore, the main implication of our results is that gait may become more unstable when the walking speed decreases. Instability could be related to a quality change in gait control which becomes less efficient with slower speed compared to preferred speed. STV reflects the control of the walking-related rhythmic stepping mechanism [2], which mainly depends on the basal ganglia and the spinal central pattern generator [1]. Low STV variability reflects the automatic processes associated with an efficient gait control and high gait safety [2,6]. An increased STV has been associated with the involvement of higher-level gait control [22], suggesting that the STV increase shown in our study could be a marker of cortical gait control. However, Dubost *et al. *[6] showed that the decrease in walking speed was an independent biomechanical factor, significantly related to an increased STV among healthy subjects performing a dual-task. An increase in STV while walking speed decreases among healthy subjects could therefore be solely related to a biomechanical feature of gait, and not necessarily to the involvement of cortical gait control.

STV assessment is a new challenge for clinicians as it provides objective, useful information for the diagnosis of gait instability [2,4,6,23]. Moreover, the recently available user-friendly portable gait analysis systems allow a simple, objective STV measurement [7,8], making the assessment of STV possible in clinical practice. However; our results suggest that a decrease in walking speed should be considered as a potential confounder while evaluating STV with the aim to diagnose gait disorders leading to instability. As example, increased STV has been associated with the efficiency of executive function [24,25]. In particular, Sheridan *et al.*. [24], and more recently Allali *et al. *[25], reported a significant relationship between a high CoV of stride time and impaired executive function among demented older adults. In both of these studies, it has been suggested that an increase in STV was an index of impaired executive function. However, none of these studies had adjusted the data for a decrease in walking speed. As consequence, we suggest that walking speed should be taken into account while exploring stride time variability in subjects with impaired executive function because increase in stride time variability could be provoked by either impaired executive function or a decrease in walking speed, or by both.

In regard to methodology, a limitation of our study could be related to the number of strides required to obtain a suitable, representative measure of gait variability. When analyzing steady-state walking across 22 m (7.32 m × 3 trials), the number of strides observed was low compared to those recommended by Owings & Grabiner, who suggested that an accurate estimation of gait variability required at least 400 steps [26]. However, previous studies recorded fewer strides than we did in our study and obtained relevant results for STV [6,9]. We therefore believe that our results are reliable as shown with trials reliability above 89%. A second limitation of our study might be linked to the repetition of trials, which may have induced motor skill learning and thus reduced STV [1,3]. The third one is the non Gaussian distribution of the variables, but the statistical procedures used are robust enough given the number of subject above 25 and the large number of steps recorded. The fourth limitation is the impossibility to generalize our results because we limited analyze to healthy young adults.

## Conclusion

We have established in the studied sample of young healthy adults a non-linear relation between STV and walking speed, whereby higher STV was reported at a slow speed, supporting the general assumption that gait could be more unstable when healthy subjects walk slower than at their normal, i.e. preferred self-selected walking speed. Furthermore, our results highlighted that a decrease in walking speed is a potential confounder while evaluating STV.

## Competing interests

The authors declare that they have no competing interests.

## Authors' contributions

OB has full access to the data in the study and takes responsibility for the integrity of the data and the accuracy of the data analyses. Study concept and design: OB, RWK and YL. Acquisition of data: YL, VD, and CA. Analysis and interpretation of data: OB, GA, FRH, VD, RWK and CA. Drafting of the manuscript: OB, GA, CA and RWK. Critical revision of the manuscript for important intellectual content: CA, FRH, GA and YL. Statistical expertise: FRH. Administrative, technical, or material support: VD and CA. Study supervision: OB and RWK.

All authors have read and approved the final manuscript

## Supplementary Material

Additional file 1 **Mean value and standard deviation of stride time parameters and stride velocity (n = 29)**. The data provided show mean value and standard deviation of stride time parameters and stride velocity (n = 29). SD: standard deviation, CoV: Coefficient of variation expressed in percentage and calculated from the formula: [(Standard deviation/Mean value) × 100]; *: Mean value of the 3 trials; †: Comparison between the 3 trials for each walking condition, based on Kruskal-Wallis test. Click here for file
