# Supplementary material for: Walking speed-related changes in stride time variability: effects of decreased speed
Source: J Neuroeng Rehabil. 2009 Aug 5;6:32. doi: 10.1186/1743-0003-6-32 (PMC2731039; doi:10.1186/1743-0003-6-32)
Supplement: Additional file 1 — Mean value and standard deviation of stride time parameters and stride velocity (n = 29). The data provided show mean value and standard deviation of stride time parameters and stride velocity (n = 29). SD: standard deviation, CoV: Coefficient of variation expressed in percentage and calculated from the formula: [(Standard deviation/Mean value) × 100]; *: Mean value of the 3 trials; †: Comparison between the 3 trials for each walking condition, based on Kruskal-Wallis test. [file 1743-0003-6-32-S1.doc]

|  | Stride time | | | | | |  | Stride velocity | |
| --- | --- | --- | --- | --- | --- | --- | --- | --- | --- |
|  | Mean value*  (ms) | P-Value† | SD*  (ms) | P-Value† | CoV*  (%) | P-Value† |  | Mean value*  (cm.s^-1^) | P-Value† |
| Preferred self-selected walking speed, mean ± SD (range) | 1082.4 ± 85.7  (959.9 – 1253.3) |  | 24.1 ± 8.8  (12.1 – 54.9) |  | 2.2 ± 0.7  (1.0 – 4.4) |  |  | 137.5 ± 16.2  (111.7 - 171.5) | 0.909 |
| % of preferred walking speed ± SD |  |  |  |  |  |  |  |  |  |
| 88.5 ± 6.5 | 1160.4 ± 114.3  (1000.7 – 1484.9) | 0.944 | 30.9 ± 12.0  (15.8 – 61.4) | 0.302 | 2.7 ± 1.0  (1.3 – 4.9) | 0.191 |  | 121.4 ± 15.4  (96.6 - 147.5) | 0.937 |
| 79.5 ± 8.9 | 1237.6 ± 144.4  (1035.1 – 1764.5) | 0.878 | 37.5 ± 16.3  (14.5 – 75.8) | 0.411 | 3.0 ± 1.2  (1.3 – 5.7) | 0.460 |  | 108.9 ± 15.5  (77.2 - 135.9) | 0.994 |
| 71.8 ± 10.7 | 1317.0 ± 188.0  (1087.1 – 2053.9) | 0.952 | 37.0 ± 17.3  (13.5 – 89.0) | 0.869 | 2.7 ± 1.0  (1.2 – 5.6) | 0.911 |  | 98.1 ± 15.7  (58.3 - 127.2) | 0.969 |
| 64.9 ± 11.5 | 1396.3 ± 225.9  (1113.9 – 2277.2) | 0.665 | 45.8 ± 23.8  (15.3 – 10.6) | 0.429 | 3.2 ± 1.5  (1.4 – 7.3) | 0.289 |  | 88.6 ± 16.4  (48.6 - 122.0) | 0.748 |
| 58.4 ± 12.7 | 1490.8 ± 278.7  (1119.7 – 2556.9) | 0.691 | 65.8 ± 39.4  (14.7 – 15.1) | 0.094 | 4.3 ± 2.4  (1.3 –10.5) | 0.266 |  | 79.8 ± 17.7  (36.5 - 117.0) | 0.776 |
| 52.5 ± 16.0 | 1641.7 ± 454.7  (1154.2 – 2975.8) | 0.837 | 75.6 ± 52.9  (13.9 – 213.4) | 0.395 | 4.3 ± 2.5  (1.2 – 10.9) | 0.400 |  | 72.2 ± 21.1  (29.7 - 109.2) | 0.922 |
| 46.1 ± 16.1 | 1843.4 ± 717.6  (1195.1 – 4525.8) | 0.898 | 84.9 ± 102.1  (21.2 – 517.9) | 0.528 | 3.9 ± 2.1  (1.7 – 11.4) | 0.695 |  | 63.3 ± 21.5  (25.8 - 104.3) | 0.894 |
| 38.9 ± 16.5 | 2138.5 ± 1138.2  (1260.4 – 6827.8) | 0.956 | 129.0 ± 151.6  (29.3 – 693.8) | 0.963 | 5.0 ± 2.8  (2.1 – 12.4) | 0.865 |  | 53.2 ± 21.6  (14.8 - 94.6) | 0.943 |
